# Textile-based radiation protection of staff during fluoroscopic guided interventions: enhancing durability, comfort and safety

**DOI:** 10.1186/s42155-025-00585-4

**Published:** 2025-08-18

**Authors:** Fredrik Gellerstedt, Petra Apell, Ziv J. Haskal

**Affiliations:** 1 Texray AB, Göteborg, Sweden; 2 Department of Technology Management and Economics, Chalmers University of Technology, Gothenburg, Sweden; 3 University of Virginia School of Medicine, Charlottesville, VA United States

## Background

Standard radiation protection materials worn by interventional and endovascular physicians are based upon metals, such as antimony (Sb), bismuth (Bi); and lead (Pb) [1]. While these rubber-like sheet products have gradually improved in less operator-borne weight, they face challenges of fractures and cracks with can compromise their shielding integrity [2]. Further, the impermeability of standard materials adds to operator discomfort due to perspiration and heat during longer procedures [3]. To address these limitations, we developed a flexible radiation protection material.

**Fig. 1:**
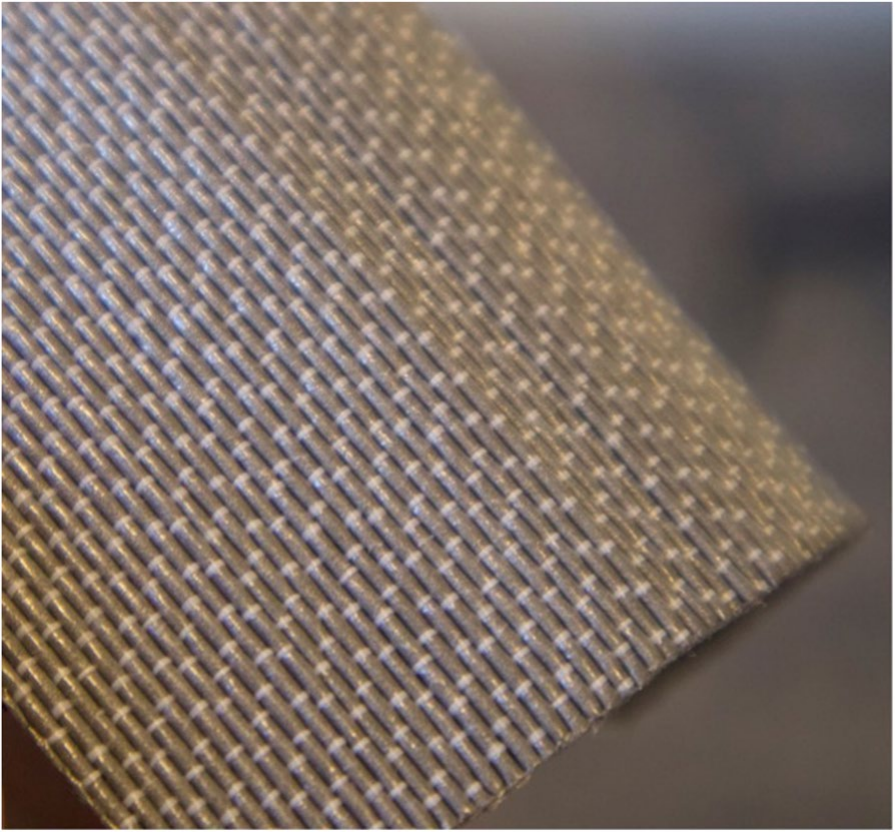
The Texray textile material, 0.25 mm Pb equivalence

This patented textile [4] is composed of a radiation-attenuating filament woven into a fabric, forming a reinforcement matrix that strengthens the material in all directions. This structure allows water vapor to pass through the weave matrix while still attenuating radiation (Fig. 1). The filaments may be filled with any attenuating metal that meets standard criteria for radiation protection and is woven in patterns designed to provide prespecified radiation protection levels. For example, the 0.25 mm lead equivalence material has a pattern designed with 17 filaments per centimeter in two layers, whereas the 0.35 mm has 25 filaments per centimeter in a three-layer weave pattern. All Texray textile attenuating materials are certified by the Personal Protection Equipment, PPE, directive (EN 2014:425) and verified by European standard EIC61331-1:2014 in tube voltage ranges from 60–150 kV. The Texray products are registered as approved medical devices by the U.S. Food and Drug Administration.

## Material durability testing

**Fig. 2:**
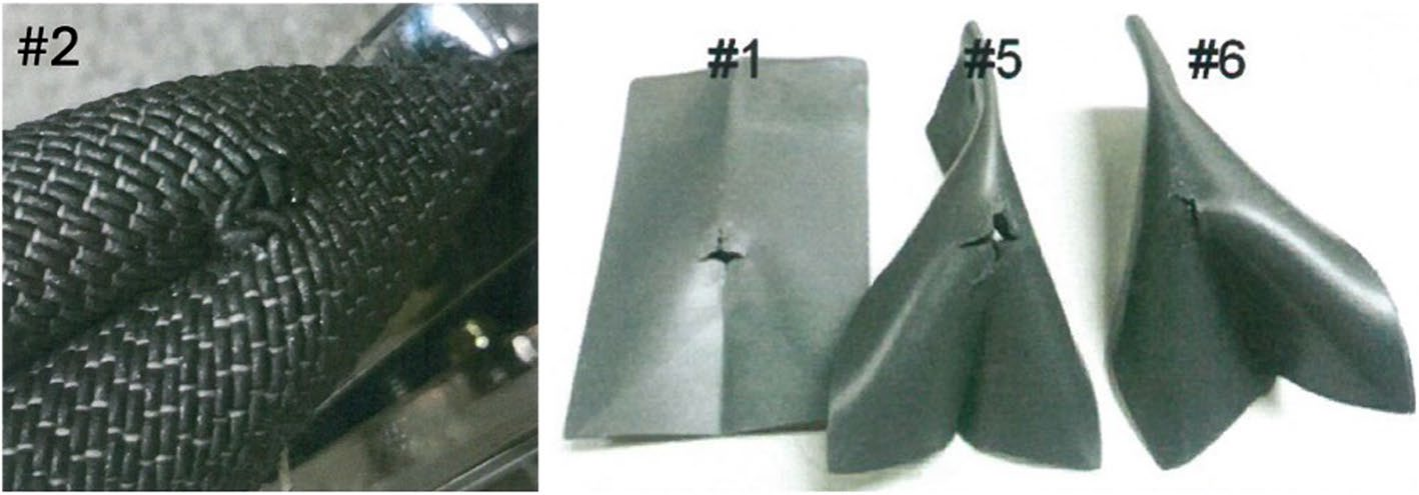
Images of Bally Flexing tester. Sample #2 is the Texray 0.25 mm Pb eq. after 300,000 cycles. The samples #1, #5 and #6 are attenuating rubber-like sheet materials after 25,000 cycles

The woven materials were tested using a Bally Leather Flexing tester (ASTM D6182-00) and compared with two attenuating rubber-like sheet materials, Xenolite by Lite Tech, and Standard Vinyl by Kemmetech. All samples, including the sheet-based materials and the Texray textile, were flexed to 300,000 cycles and visually inspected for damage. Damage was assessed by grading after specific number of cycles: grade 1, no effect/intact; grade 2, marked or severe creasing or other negative visual effects; grade 3, slight cracking of coating and/or material; grade 4, marked cracking; grade 5, severe cracking. Testing results demonstrated that both materials of standard rubber-like sheet materials showed grade of 3 effects (slight cracking) at 10,000 cycles and grade 5 (severe cracking) at 25,000 cycles. The Texray textile material showed grade of 3, slight cracking, at 300,000 cycles. Assuming that slight cracking is the starting point for pinholes and tear, the textile material showed 30 times better durability in folding and flexing compared to the sheet-based materials (Fig. 2).

## Breathability & comfort

To assess breathability, air permeability was evaluated in accordance with SS-EN 139:2025. The Texray 0.25 mm lead equivalent and 0.35 mm lead equivalent materials showed a permeability of 6 mm/s, or 36 L of air per dm^2^ per minute, at a pressure drop of 100 Pa. In contrast, the standard rubber-like radiation attenuating material was completely impermeable to air.

## Use cases

**Fig. 3:**
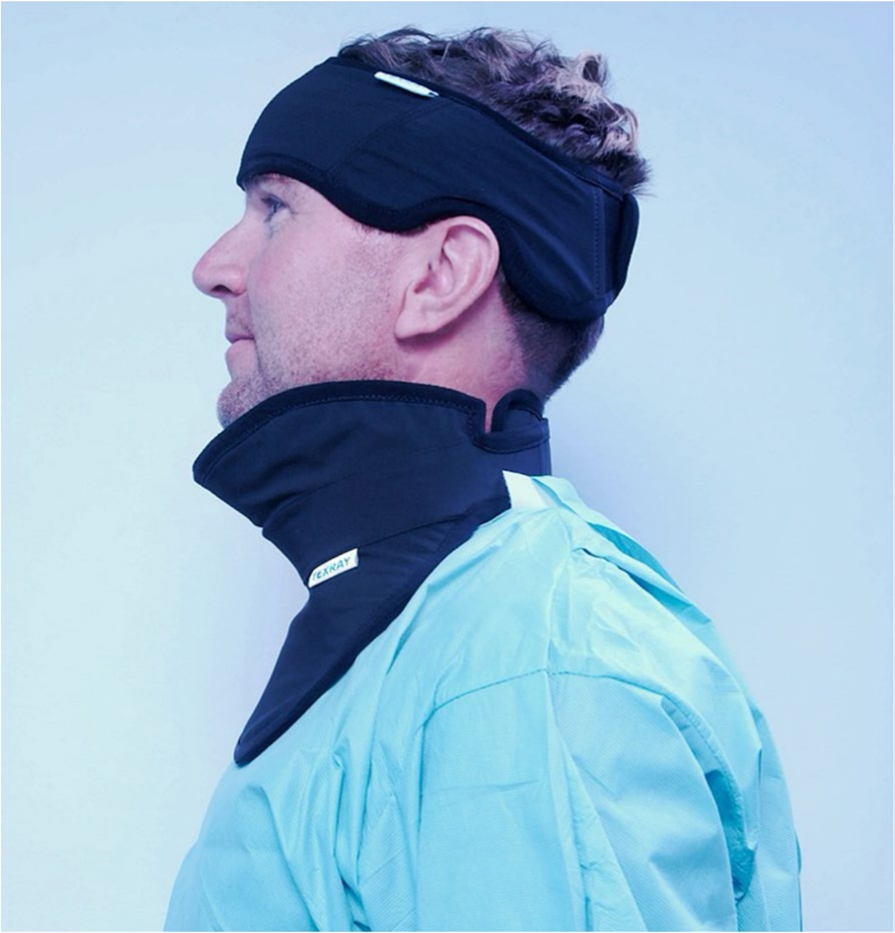
Neck, Face and Head Protector, HeadPeace and MindPeace, from Texray AB, engineered from Texray textile material

The durability, flexibility, and breathability of the material allows expansion of personnel protection with mission-specific designs, such as ones to improve neck and jaw protection and cranial vault and brain, by creating three-dimensional custom designs to maximize operator coverage. The MindPeace and HeadPeace products represent two such examples—designed to both better cover the thyroid gland in all sized operators and expand upward scatter protection to the jaw and lower face (Fig. 3). Used together, these shields provided up to 90% additional radiation protection of the neck, face and head when compared to standard thyroid collars [5]. Clinical evaluations demonstrated an attenuation efficiency of 75.5–96.7% depending on the product and operational area [6]. Moreover, these products have been proved more comfortable compared to products made of sheet-based materials [7].

**Fig. 4:**
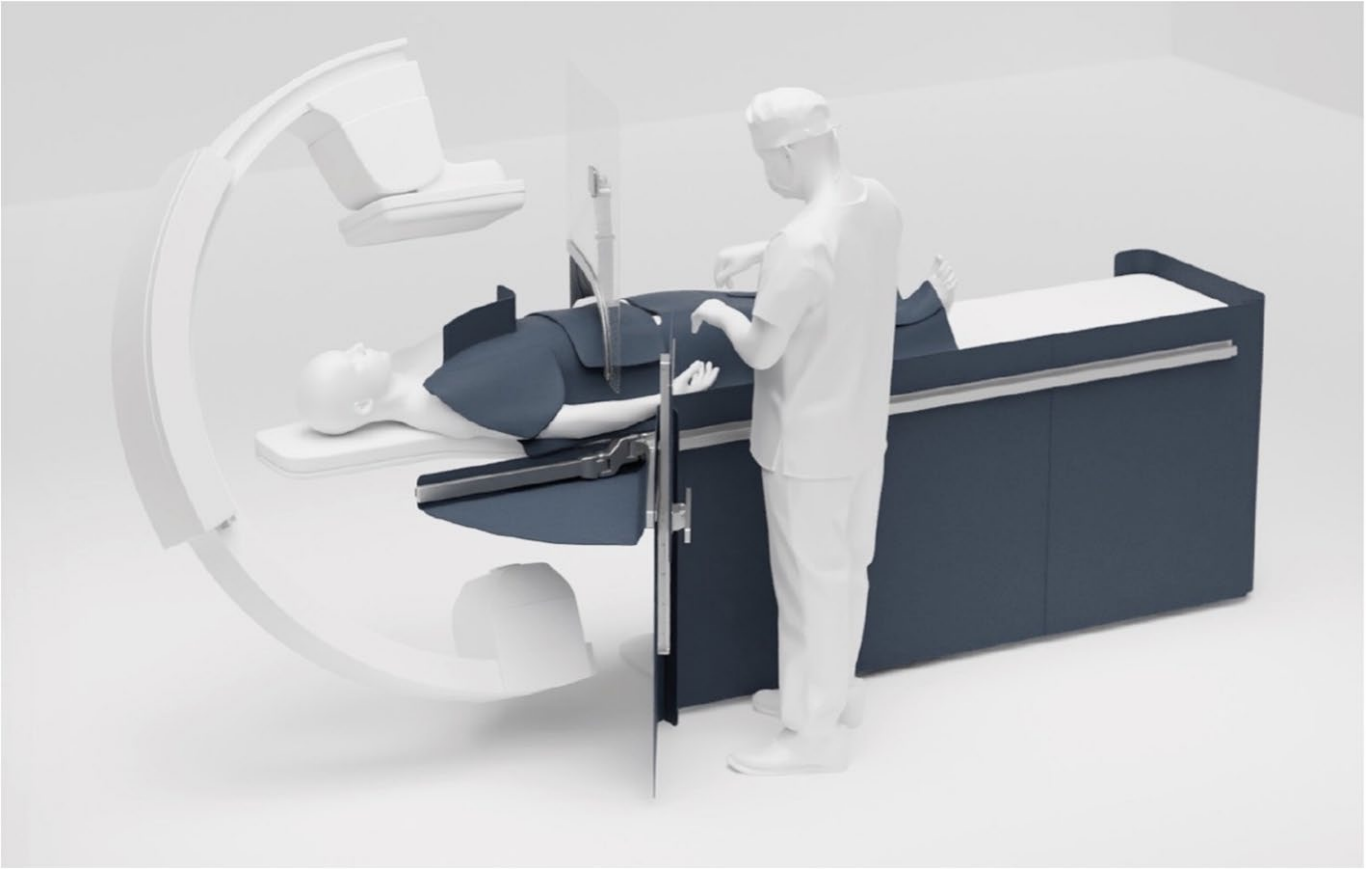
The newly invented MasterPeace ERPD, engineered from Texray textile material

The durability of the Texray textile makes it possible to fold radiation shields without risking radiation protection failure, allowing new applications to be designed. One such example is the tableside Enhanced Radiation Protection Device (ERPD), “MasterPeace” (Fig. 4), which is designed to protect all room staff without restricting patient access during cases and without restricting full patient access during emergent conditions.

## Discussion

Durability and flexibility are critical parameters for well-functioning radiation protection materials—the extensive flex testing showed that standard materials can develop severe cracking after only 25,000 cycles. The data confirms a 4-year longitudinal follow-up study affirming the fracture risks of existing personal radiation protective equipment [2]. Up to 50% of the evaluated products showed tears and cracks; 6% of these showed tears within the first year of use. In contrast, the woven material proved far less likely to crack and tear, suggesting far greater durability and safety over sheet-based materials. This can translate to both greater assurance of uninterrupted protection for radiation workers and potentially less apron-screening for craft detection and replacement.

The flexibility of the woven material allows design of site-specific protection, which could expand routine protection of underrecognized areas, exemplified by the head, neck and face protection and circumferential table solutions that could expand protection to all adjacent room personnel, including technologists, nurses, and anesthesiologists. Further applications could expand to breathable PPE body garments, covering upper arms and shin guards.

In summary, the Texray attenuating textile provides improved material properties to overcome the material challenges related to durability, comfort and safety of radiation protection devices.

## Data Availability

The referred data are available from the corresponding author on reasonable request.
